# Family factors associated with dental caries among 5-year-old preschool children

**DOI:** 10.3389/fdmed.2024.1473194

**Published:** 2025-01-20

**Authors:** Giovanna Torqueto Castilho, Marília Narducci Pessoa, Caroline Correa de Oliveira, Letícia Santos Alves de Melo, Elaine Pereira Silva Tagliaferro, Vanessa Pardi

**Affiliations:** ^1^ Department of Morphology and Children's Clinic, São Paulo State University (UNESP), School of Dentistry, Araraquara, Brazil; ^2^ Department of Community Dentistry, São Paulo State University (UNESP), School of Dentistry, Araraquara, Brazil; ^3^ School of Dental Medicine, East Carolina University, Greenville, NC, United States

**Keywords:** dental caries, family, oral health perception, oral health, toothbrushing

## Abstract

**Background:**

Although dental caries is largely preventable, it remains highly prevalent among children.

**Aim:**

Evaluate family factors associated with the prevalence of dental caries in 5-year-old children.

**Design:**

This cross-sectional study recruited 5-year-old children from public preschools in Araraquara, São Paulo, Brazil. Data on sociodemographic factors, family routines, and oral health practices were gathered via a self-administered questionnaire completed independently by caregivers. Children received a dental clinical examination at school and caries experience was recorded using the dmft and SiC (Significant Caries index) indexes following the World Health Organization (WHO) criteria.

**Results:**

Data analysis was performed using Chi-square and multiple logistic regression using a significance level of 5%. Of the 578 children in the study, 67.3% were caries-free. The mean dmft index was 1.14 (SD = 2.24), while the SiC index stood at 3.46 (2.69). Factors such as income, caregiver assistance with toothbrushing, caregiver perception of child and parent oral health, and prioritization of dental visits within the family were associated with caries presence.

**Conclusions:**

Overall, dental caries prevalence was low in this population, with family factors demonstrating significant associations with dental caries. The attitudes of caregivers regarding oral health appear to wield considerable influence over the dental health of their children.

## Introduction

Dental caries is the most prevalent non-communicable chronic disease in the world, according to the World Health Organization (1). The Brazilian National Oral Health Survey (SB BRASIL) conducted in 2010 shows that dental caries affects approximately 53.4% of 5-year-old children (2). Dental caries is a chronic multifactorial disease that develops through a dynamic interaction among tooth structures, biofilm, and sugars and it involves a continuous process of demineralization and remineralization of the tooth surface. When demineralization persists over time, carious lesions begin to form (3). In addition to factors such as poor dietary habits and oral hygiene, another predisposing factor contributing to the increase of dental caries in preschool children is maternal attitudes and knowledge about oral health (4–6). Fisher-Owens et al. in 2007 (7) proposed a conceptual model of factors influencing children's oral health: individual factors such as health behavior and practices, dental visits, physical and demographic attributes, for example; family factors such as socioeconomic status, parents' health status, social support, family composition and culture, among others. Furthermore, community factors such as the availability of dental and health services, social capital, and the overall social environment are also integral components of the model (7).

A recent systematic review of parental factors influencing the presence of dental caries in early childhood in developing countries suggests that income, education, knowledge of oral health, and attitudes are associated with the prevalence of this disease (8). Low parental literacy has been correlated with adverse outcomes in children's oral health. Furthermore, it is associated with practices that increase the risk of dental caries, including the consumption of cariogenic foods and beverages, inadequate use of fluoride toothpaste, and insufficient brushing habits (9–11).

During the preschool age, parents play a crucial role as mediators of information, behaviors, and decisions related to their child's health. Psychosocial factors, such as resilience and self-efficacy, emerge as significant mediators influencing oral health behaviors, including the pivotal decision to seek dental care (12, 13). Exploring actions and factors linked to parents' perceptions of their children's oral health is essential for identifying key indicators that may influence behaviors and approaches related to oral health maintenance. This understanding can directly impact practices like consistent diet control and the proactive pursuit of dental care. Therefore, the aim of this study was to evaluate family-related factors contributing to the prevalence of dental caries among 5-year-old children.

## Materials and methods

### Study design

This cross-sectional study received approval from the Research Ethics Committee of the São Paulo State University (UNESP) School of Dentistry, Araraquara (FOAr-UNESP (CAAE 62756922.0.0000.5416) and authorization from the Department of Education Araraquara, São Paulo, Brazil. Prior to data collection, all participants provided signed informed consent and assent forms.

### Setting and participants

Data collection was conducted between June and December 2023 at Education and Recreation Centers (ERCs) situated in the municipality of Araraquara, located in the state of São Paulo, Brazil. A total of forty-five ERCs were identified through the Educational Portal of the City Hall of Araraquara. Among these centers, only three did not participate in the project, and one was not invited due to the absence of enrolled 5-year-old children. The study included 2,401 children. Inclusion criteria for participation comprised: (a) children aged 5 years; (b) mothers who were 18 years old or older (c) appropriately authorized informed consent forms by the caregivers; and (d) properly authorized assent forms by the children. A total of 578 pairs of participants (children and caregivers) returned the consent.

### Data collection

Data collection encompassed the dissemination of semi-structured and self-administered questionnaires by the school to caregivers, supplemented by digital formats accessible through Google Forms. The questionnaires covered socioeconomic and demographic data, family routines, utilization of dental services, oral health-related inquiries, knowledge level on oral health, and information pertaining to the child's oral health. Questions within the questionnaire were derived from validated instruments (Supplement). Upon the completion of the questionnaires by caregivers, a clinical examination of the children was conducted, after their authorization through the Assent and Consent Form.

### Clinical examination

Three examiners conducted the examinations after 20 h of training by two gold-standard examiners (median agreement rate of 85%). The training included theoretical and practical calibration sessions, using clinical case photographs and discussions to reach diagnostic consensus.

The questionnaire underwent a pre-testing phase involving 11 mothers and/or caregivers of children aged between 4 and 12 years, who were patients at the Dental Public Health Clinic of the School of Dentistry, Araraquara and presented with similar sociodemographic characteristics. During this phase, the researcher (GTC) monitored the response duration, observing an average completion time of 15 min. Following questionnaire completion, participants expressed uncertainties regarding specific questions. Subsequent adjustments were made to improve the questionnaire's clarity and suitability for participants.

The oral clinical examinations were performed in schools, under natural light, using clinical mirror and CPI probe. Visual-tactile methods were employed during the examinations. In adherence to COVID-19 protocols at the schools, there was no prior brushing before the examination. Dental caries data were collected during the examination following the criteria outlined in SB BRASIL 2020 and WHO 2013 (dmft index). One examiner conducted the child's oral examination and verbally dictated the codes, while another examiner recorded the codes on paper forms.

### Statistical analysis

Descriptive analyses, *χ*^2^ tests, and two multiple logistic regression models were utilized for data analysis. The data for the dmft index and questionnaire were dichotomized at the median. Questions on last dental visit and assistance with toothbrushing were not dichotomized because we aimed to highlight the positive categories (assistance with tooth brushing) in comparison to the negative category (no assistance). Questionnaire data from children that did not receive a clinical exam was excluded from the analysis. The dmft index and the SiC index (Significant Caries Index) (14) were computed, with the SiC index representing the average of the dmft index calculated for one-third of the population with the highest dental caries prevalence. Variables with a *P*-value < 0.20 in the univariate analysis were included in the logistic regression models. This liberal *P*-value threshold increases the likelihood of retaining important predictors in the final model (15–17). Data analysis was performed using SPSS software (version 28.0.0.1), with a significance level set at 5%.

## Results

The study sample comprised of 578 pairs of participants (children and caregivers). The mean dmft index was 1.14 (SD = 2.24), while the SiC index was at 3.46 (SD = 2.69). The prevalence of dental caries experience was 32.7%.

Table 1 presents the results categorized according to the conceptual model of Fisher-Owens et al. 2007 (7), including child, family, and community levels. Among children with dental caries experience (dmft ≥ 1), 36.7% had not visited the dentist for more than 2 years. The majority (67.7%) of children whose parents reported daily tooth brushing habits remained free of dental caries. In contrast, children who consumed sugary foods and drinks three or more times a day exhibited a dental caries prevalence of 31.7%, while the majority (68.3%) of those consuming such items up to twice a day remained caries-free.

**Table 1:** Association between factors related to children, families, and communities and the presence or absence of dental caries in 5-year-old children. (*N* = 578). Araraquara, São Paulo, Brazil (2023).

Variables	Total *N* (%)	dmft = 0 *N* (%)	dmft ≥ 1^a^ *N* (%)	*P*-value
Child Factors				
Gender				
Female	286	198 (69.2%)	88 (30.8%)	0.328
Male	292	191 (65.4%)	101 (34.6%)	
Child's Last Dental Visit				
Up to 2 years ago	300	190 (63.3%)	110 (36.7%)	0.122
More than 2 years ago	35	26 (74.3%)	9 (25.7%)	
Never/Don't know	233	165 (70.8%)	68 (29.2%)	
Dental Insurance				
Yes	436	286 (65.6%)	150 (34.4%)	0.402
No	133	95 (71.4%)	38 (28.6%)	
Don't know	2	1 (50.0%	1 (50.0%)	
Daily Tooth Brushing				
Yes	526	356 (67.7%)	170 (32.3%)	0.464
No	48	30 (62.5%)	18 (37.5%)	
Consumption of Sweet Foods/Beverages (daily frequency)				
Up to 2×/day	375	256 (68.3%)	119 (31.7%)	0.377
≥3×/day	192	124 (64.6%)	68 (35.4%)	
Family Factors				
Education				
Up to high school (incomplete)	408	272 (66.5%)	137 (33.5%)	0.488
Completed high school/incomplete higher education/completed higher education	164	114 (69.5%)	50 (30.5%)	
Marital Status				
Single/Divorced/Widowed	240	160 (66.7%)	80 (33.3%)	0.744
Married/Common-law marriage	334	227 (68.0%)	107 (32.0%)	
Self-rated Oral Health perception				
Good/Excellent	340	242 (71.2%)	98 (28.8%)	0.023
Fair/Poor/Very Poor	235	146 (62.1%)	89 (37.9%)	
Parent’s rating of child’s oral health				
Excellent/Good	365	284 (77.8%)	81 (22.2%)	<.001
Fair/Poor/Very Poor	208	100 (48.1%)	108 (51.9%)	
Assists in tooth brushing				
No	67	35 (52.2%)	32 (47.8%)	0.010
At least 1×/every day	430	302 (70.2%)	128 (29.8%)	
At least 1×/several times a week	76	48 (63.2%)	28 (36.8%)	
White spot as first sign of dental caries				
Yes	496	325 (65.5%)	171 (34.5%)	0.145
No	53	40 (75.5%)	13 (24.5%)	
Sugary foods/drinks cause dental caries				
Yes	542	364 (67.3%)	178 (32.8%)	0.187
No	17	14 (82.4%)	3 (17.6%)	
Last Visit to Dentist by caregiver				
Up to 1 year ago	357	249 (69.7%)	108 (30.3%)	
More than 1 year ago	173	108 (62.4%)	65 (37.6%)	0.227
Never/Don't know	40	28 (70.0%)	12 (30.0%)	
Monthly Income				
≤R$3.000 (≤US$600)	279	168 (60.2%)	111 (39.8%)	<.001
>R$3.000 (>US$600)	174	131 (75.3%)	42 (24.7%)	
Number of Rooms in Residence				
Up to 2	410	270 (65.9%)	140 (34.1%)	0.139
> 2	159	115 (72.3%)	44 (27.7%)	
Number of Residents				
Up to 4	425	294 (69.2%)	131 (30.8%)	0.117
>4	148	92 (62.2%)	56 (37.8%)	
Number of Residents under 18 years old				
Up to 2	452	311 (68.8%)	141 (31.2%)	0.049
>2	115	68 (59.1%)	47 (40.9%)	
Family meals together				
Every day	350	242 (69.1%)	108 (30.9%)	0.199
0–6 days	222	142 (64.0%)	80 (36.0%)	
Dental visit is a priority				
Yes	529	363 (68.6%)	166 (31.4%)	0.023
No	46	24 (52.2%)	22 (47.8%)	
Community Level				
Neighborhood				
Very Safe/Moderately Safe	319	221 (69.3%	98 (30.7%)	0.645
Neither Safe nor Unsafe/Moderately Unsafe/Very Unsafe	240	162 (67.5%)	176 (31.5%)	
Neighborhood Amenities (playground, park)				
Yes	347	240 (69.2%)	107 (30.8%)	0.241
No	228	147 (64.5%)	81 (35.5%)	

^a^ Event of interest.

At the family level, when caregivers rated their own oral health as good/excellent, 71.2% of their children were caries-free. Additionally, children whose caregivers rated their oral health as good/excellent showed no dental caries (77.8%). Children whose parents who did not assist them with tooth brushing displayed dmft ≥ 1 (47.8%). Children from households with an income > R$3,000 (>US$600) showed better oral health, with 75.3% remaining cavity-free. Caregivers who stated that oral health was *a priori*ty exhibited better oral conditions for their children, with 68.6% being cavity-free and only 31.4% experiencing dental caries.

At the community level, 69.3% of children whose caregivers felt safe/moderately safe in their neighborhoods did not have dental caries. Most children whose parents reported that their neighborhood had playgrounds and parks did not have dental caries (69.2%).

Variables with *p* < 0.20 in the univariate analysis were included in the logistic regression models. The first model was adjusted for child's last dental visit, self-rated oral health perception, Parent's rating of child's oral health, assistance with toothbrushing, income, number of rooms in the residence, number of residents, number of residents under 18 years old, family meals, dental visit as a priority (Table 2). In the second model, besides the variables described before, we included the ones related to knowledge of dental caries (White spot as first sign of dental caries, Sugary foods/drinks cause dental caries) (Table 3). Among these variables, family factors such as income, involvement in child tooth brushing, perception of the child's oral health, and prioritization of dental treatment within the family, were found to be associated with the occurrence of dental caries. However, when variables related to knowledge of dental caries (recognizing the first signs of dental caries and understanding if sugary drinks/food cause the dental caries) were included in the logistic model construction, income was no longer retained in the final model, while perception of the parent's own oral health remained significant (Tables 2, 3).

**Table 2:** Multiple logistic regression analysis for the absence/presence of dental caries (dmft). Araraquara, São Paulo, Brazil (2023).

Variables	dmft index		AOR	CI 95%	*P*-value
	dmft = 0	dmft ≥ 1^a^			
Monthly Income					
≤R$3.000 (≤US$600)	168 (60.2%)	111 (39.8%)	1.736	1.060–2.849	0.028
>R$3.000 (>US$600)	131 (75.3%)	42 (24.7%)	Ref		
Assists in tooth brushing					
No	35 (52.2%)	32 (47.8%)	Ref		
At least once daily	302 (70.2%)	128 (29.8%)	0.485	0.253–0.931	0.030
At least once a few times a week	48 (63.2%)	28 (36.8%)	0.431	0.186–0.997	0.049
Parent's rating of child's oral health					
Excellent/Good	284 (77.8%)	81 (22.2%)	Ref		<0.001
Fair/Poor/Very Poor	100 (48.1%)	108 (51.9%)	3.895	2.444–6.208	
Dental visit is a priority					
Yes	363 (68.3%)	166 (31.4%)	Ref		0.036
No	24 (52.2%)	22 (47.8%)	2.183	1.050–4.524	

Ref, reference category for independent variable; AOR, Adjusted odds ratio; CI, Confidence interval.

^a^ Event of interest.

**Table 3:** Multiple logistic regression analysis for absence/presence of dental caries (dmft) including variables related to knowledge about dental caries. Araraquara, São Paulo, Brazil (2023).

Variables	dmft index		AOR	CI 95%	*P*
	dmft = 0	dmft ≥ 1^a^			
Assists in tooth brushing					
No	35 (52.2%)	32 (47.8%)	Ref		
At least once daily	302 (70.2%)	128 (29.8%)	0.457	0.233–0.895	0.023
At least once a few times a week	48 (63.2%)	28 (36.8%)	0.401	0.169–0.953	0.038
Parent's rating of child's oral health					
Excellent/Good	284 (77.8%)	81 (22.2%)	Ref		<0.001
Fair/Poor/Very Poor	100 (48.1%)	108 (51.9%)	4.278	2.635–6.96	
Self-rated Oral Health perception					
Good/Excellent	242 (71.2%)	98 (28.8%)	Ref		0.047
Fair/Poor/Very Poor	146 (62.1%)	89 (37.9%)	1.661	1.006–2.739	
Dental visit is a priority					
Yes	363 (68.6%)	166 (31.4%)	Ref		0.022
No	24 (52.2%)	22 (47.8%)	2.392	1.135–5.025	

Ref: reference category for independent variable. AOR: Adjusted Odds Ratio. CI: Confidence Interval.

^a^ Event of interest.

## Discussion

According to the World Health Organization (WHO), dental caries affects approximately 2.3 billion people worldwide, with 53.4% of 5-year-old children in Brazil affected (2, 18). Beyond environmental factors, parental influences play a significant role in shaping children's oral health (8).

Our study findings revealed that familial factors, including income, involvement in children's dental brushing, perception of oral health, and prioritization of dental visits, significantly influence the presence of dental caries in preschool children.

Socioeconomic status plays a crucial role in dental caries, as evidenced in this study, where children from lower-income families were more likely to develop dental caries. This finding aligns with a study conducted in 2022 by Sultana et al., which found a significant association between low family income and the caries index, with these children being 4.75 times more likely to experience the disease (19). Additionally, association of income trajectories and dental caries was found in birth-cohort studies, with poverty at birth and throughout life being linked to higher numbers of decayed teeth (20, 21).

Socioeconomic status can indeed serve as a barrier to acquiring health knowledge. A recent study concluded that mothers with low income, who have children aged 3–5 years, often exhibit inadequate preventive behaviors for caries and fail to adopt sufficient practices to ensure their children's oral health (22). In a study conducted in Araçatuba, São Paulo, in 2010, the dmft index for 5-year-old children was 1.65 (SD = 2.67), with dental caries being significantly more prevalent in low-income children (23). In the present study, the dmft index found for 5-year-old children was 1.14 (SD = 2.24), and a higher proportion of children from lower-income families presented dental caries, consistent with the findings of previous studies. Interestingly when variables related to knowledge of dental caries were included in the logistic model, income did not remain a significant factor. Instead, parent's perception of their own oral health emerged as influential factor, together with involvement in child tooth brushing, parent's perception of child's oral health, and prioritization of dental treatment. These results suggest that other factors, that surpass socioeconomic factors, may play a role on dental caries experience, for example oral health literacy, attitudes, and practices in determining caries risk. The common risk approach shows that oral diseases share common risk condition with other chronic noncommunicable diseases (diabetes, obesity, stroke, i.e.,) (24). Living in unhealthy conditions may determine risk behaviors (25).

Ensuring proper dental brushing practices is crucial for maintaining good oral health, especially among children. Supervised brushing is particularly important for preschoolers, as their self-care abilities are still developing, resulting in poor quality brushing and sometimes incorrect techniques (26). The age at which children commence brushing, the frequency of brushing, and whether parents assess the effectiveness of their children's brushing directly influence the prevalence of early childhood caries (ECC) (27). In this study, children who received parental assistance during brushing demonstrated a reduced risk of developing dental caries, even when this assistance occurred only once a day on certain days of the week.

In a study aiming to quantify the association between caregivers' perception of their own oral health and that of their child, the authors noted a significant correlation between the caregivers' poor perception of their own oral health and that of their child (28). Similarly, in our study, caregivers demonstrated notable accuracy in assessing their child's oral health and current oral condition. Children whose caregivers reported a negative perception of their children's oral health exhibited a higher likelihood of experiencing dental caries.

Children whose caregivers did not prioritize a dental visit were approximately twice as likely to experience dental caries. During childhood, parents play a pivotal role in decision-making and shaping their child's habits, particularly concerning their oral health and dental care visits. Tooth brushing habits from parents that do not present depression reciprocally affect child's tooth brushing behaviors (29), as well as parent's tooth brushing behavior affects the tooth-brushing habits for their children (30). The education on prevention and oral hygiene of parents influence the oral health of children mainly when they receive individualized oral health education (31, 32). Oral health education should not be the sole responsibility of oral health care providers; instead, a collaborative effort among professionals from various fields may improve oral health in children and the general population (31, 33). To support this, oral health should be integrated into their curricula (34).

Several factors may contribute to parents not taking their children to the dentist. These reasons could include concerns about the child's behavior during dental visits, socioeconomic constraints due to the expenses associated with treatment, insufficient knowledge and awareness about oral health, and prevalent myths and misconceptions regarding oral health. One such misconception is the belief that baby teeth inherently do not necessitate treatment, as they are expected to shed naturally (35–37).

Parents may refrain from taking their children to the dentist for various reasons, including past negative experiences, psychological barriers, fear of pain, anxiety, and a lack of trust in dental professionals. However, the presence of a supportive and knowledgeable dental professional can play a crucial role in addressing these concerns, instilling confidence in parents and making them more inclined to seek dental care for their children when necessary (38). In addition to the reassurance provided by dental professionals, there is a pressing need to prioritize educational efforts aimed at both parents and children. By emphasizing the importance of oral health and providing motivation and encouragement, dental providers support caregivers to actively engage in their children's oral hygiene routines (39). Educational programs tailored to sensitize caregivers to the significance of dental care can equip them with the necessary information and resources to promote better oral health practices in their households. Ultimately, by fostering a collaborative approach that prioritizes education and support, we can strive to improve the oral health outcomes of children and alleviate barriers to accessing dental care.

Dental caries, a complex condition influenced by various factors, encompasses individual, familial, and community dynamics that contribute to its occurrence. While our study explores these factors, community-level variables did not emerge as significant predictors of dental caries presence. Interestingly, our findings revealed that most caregivers expressed a sense of security within their neighborhoods. Furthermore, they reported ample social interaction opportunities for children, such as playgrounds and parks. Despite these positive community aspects, the study did not uncover a significant association with the prevalence of dental caries. This suggests that while community factors play a role in shaping oral health outcomes, other individual and familial determinants may exert a more substantial influence. Further exploration is warranted to fully understand the interplay of these factors in the development of dental caries and to inform targeted intervention strategies.

This study presents some limitations. Firstly, the use of a self-administered questionnaire introduced the possibility of incomplete responses or multiple selections for questions requiring single responses. Additionally, the non-participation of all schools, coupled with the exclusive inclusion of public schools, poses a limitation. This selective sampling approach may limit the generalizability of findings, as the sample of 5-year-old children may not fully represent the broader population within the city, state, or country. Nevertheless, it is noteworthy that our study encompassed a substantial sample size of 578 5-year-old children, enabling robust statistical analyses with a high level of test power (99%). Despite the acknowledged limitations, the extensive sample size enhances the reliability of our findings and contributes valuable insights into the prevalence and determinants of dental caries among young children.

Family relationships play a pivotal role in fostering overall health and well-being, alongside the connections forged within the school environment and the broader community. Acknowledging the importance of these interconnected social networks, dental professionals serve as catalysts in establishing communication channels within communities. By engaging with social agents, dental professionals contribute to collective efforts in prioritizing and advancing oral health initiatives, ultimately benefiting individuals and families. However, to achieve significant oral health gains, these strategies should be complemented by a common risk approach within the community (25) and policies targeting social determinants of health (40).

## Conclusion

In this population, a low prevalence of dental caries was observed, with familial factors showing an association with this condition. The attitudes of caregivers regarding oral health seem to exert a significant influence on the dental health of their children. Consequently, dental professionals are tasked with assuming an educational, motivational, and positive reinforcement role to cultivate attitudes conducive to improving and maintaining oral health. Through these efforts, dental professionals can contribute to shaping a family environment that prioritizes oral health promotion.

## Data Availability

The raw data supporting the conclusion will be made available by the authors upon request, without undue reservation.
